# The Effect of COVID-19 on Training and Case Volume of Vascular Surgery Trainees

**DOI:** 10.1177/1538574420985775

**Published:** 2021-01-11

**Authors:** Nicole Ilonzo, Issam Koleilat, Vivek Prakash, John Charitable, Karan Garg, Daniel Han, Peter Faries, John Phair

**Affiliations:** 1 Division of Vascular Surgery, Department of Surgery, The Icahn School of Medicine at Mount Sinai, New York, NY, USA; 2 Division of Vascular and Endovascular Surgery, Montefiore Medical Center/Albert Einstein College of Medicine, Bronx, NY, USA; 3 Division of Vascular and Endovascular Surgery, New York University School of Medicine, New York, NY, USA

**Keywords:** education, COVID-19, vascular training

## Abstract

**Background:**

In many facilities, the coronavirus disease (COVID-19) pandemic caused suspension of elective surgery. We therefore sought to determine the impact of this on the surgical experience of vascular trainees.

**Methods:**

Surgical case volume, breadth, and the participating trainee post-graduate level from 3 large New York City Hospitals with integrated residency and fellowship programs (Mount Sinai, Montefiore Medical Center/Albert Einstein College of Medicine, and New York University) were reviewed. Procedures performed between February 26 to March 25, 2020 (pre-pandemic month) and March 26 to April 25, 2020 (peak pandemic period) were compared to those performed during the same time period in 2019. The trainees from these programs were also sent surveys to evaluate their subjective experience during this time.

**Results:**

The total number of cases during the month leading into the peak pandemic period was 635 cases in 2019 and 560 cases in 2020 (12% decrease). During the peak pandemic period, case volume decreased from 445 in 2019 to 114 in 2020 (74% reduction). The highest volume procedures during the peak pandemic month in 2020 were amputations and peripheral cases for acute limb ischemia; during the 2019 period, the most common cases were therapeutic endovascular procedures. There was a decrease in case volume for vascular senior residents of 77% and vascular junior and midlevel residents of 75%. There was a 77% survey response rate with 50% of respondents in the senior years of training. Overall, 20% of respondents expressed concern about completing ACGME requirements due to the COVID-19 pandemic.

**Conclusions:**

Vascular surgery-specific clinical educational and operative experiences during redeployment efforts have been limited. Further efforts should be directed to quantify the impact on training and to evaluate the efficacy of training supplements such as teleconferences and simulation.

## Introduction

The coronavirus disease (COVID-19) pandemic has led to a major change in the world. As of October 1st, 2020, there were over 7 million cases of coronavirus in the United States.^1^ New York experienced 460,000 cases and 32,757 deaths.^1^ Hospitals overwhelmed with COVID cases halted elective surgeries.^2^ New York City rapidly became one of the global epicenters of the pandemic, with the number of COVID-19 patients hospitalized in 1 hospital system alone in excess of 2,000 patients in the peak of the pandemic.^3^

Many professionals, surgeons, nurses and other surgical staff were reassigned and deployed to different units as an embargo on elective surgery was put in place. Surgeons and surgical trainees were asked to function as intensivists, emergency room physicians, and more. Medical workers were deployed to various sites to function in extended roles.

The COVID-19 pandemic also impacted medical education and training.^4^ Medical students in their final year graduated early to join the workforce and assist in care of COVID-19 patients.^5^ Residents and fellows in varying fields such as plastic surgery^6^ and urology^7^ reported a significant reduction in their usual clinical and surgical activities. Interventional radiology residencies and fellowships saw a change in the case numbers, particularly in the number of breast imaging and nuclear medicine studies reviewed.^8^

On March 17, 2020, the Association of American Medical Colleges (AAMC) released a recommendation in support of pausing all clinical activities for medical students.^9^ The Accreditation Council for Graduate Medical Education (ACGME) subsequently released guidelines regarding training in the face of the pandemic. They defined 3 stages: Stage 1, “business as usual”; Stage 2, “increased but manageable clinical demand”; and Stage 3, “routine care education and delivery must be reconfigured to focus only on patient care” due to the overwhelming volume of COVID-19 patients.^10^ For Stage 3, there were no formal recommendations for teaching activities in the core specialty except to ensure that trainees were well-equipped and supervised in pandemic-related tasks. We therefore sought to determine the effect of the COVID-19 pandemic on vascular surgical education during the height of the pandemic at 3 major teaching institutions in one of the global epicenters.

## Methods

Three tertiary, academic New York City hospitals with integrated residency and fellowship programs (Mount Sinai Medical Center, Montefiore Medical Center, and New York University) were included. All cases performed between February 25, 2020 and April 25, 2020 were evaluated and compared to cases performed during the same time period in 2019. The time period was divided into the pre-pandemic (PRE) period (February 26 to March 25) and the peak pandemic (PPP) period (March 26 to April 25). The PPP most closely coincided with Stage 3 of the ACGME Pandemic Emergency Status Guidance; elective cases were canceled during the majority, if not all, of this time period. Cases were categorized according to the ACGME classification system,^11^ which includes either “Defined Category,” “Area,” or “Type.” In this study, cases were characterized by ACGME defined category or area. This included abdominal, amputation, cerebrovascular, endovascular aneurysm repair, endovascular diagnostic, endovascular therapeutic, peripheral, vascular access, and venous categories. Case volume and breadth and the participating trainee post-graduate level were compared.

Additionally, a brief adjunctive survey of trainees in these hospital systems was performed. Survey questions included the nature of any redeployment, time out of work related to personal COVID-19 illness, post-graduate year (PGY), utilization of surgical simulation training, and concerns regarding meeting case volume requirements (Table 1). The survey was anonymous and was distributed and administered electronically.

**Table 1.** Survey Questions and Answer Options.

Question Number	Question	Answer options/comments
1	What is your clinical postgraduate year?	Options: PGY-1 to PGY-7
2	Have you met your minimum case numbers for ACGME required core cases?	Options: Yes / No
3	Have you been reassigned to a medical unit during the COVID-19 pandemic? If so which unit?	Options: Yes, ICU / Yes, Emergency Room / Yes, Medical floor / Yes, other / No
4	Have you been involved in any emergency surgeries during the COVID-19 pandemic?	Options: Yes / No
5	Have you been ill or out of work during the COVID-19 pandemic?	Options: Yes, non-COVID related / Yes, COVID-related / No
6	If you have been out of work during the pandemic, for how long?	Options: 1 day / 2-5 days / 7 days / 2 weeks / Greater than 2 weeks / Not out of work
7	Have you been involved in line placement during the COVID-19 pandemic?	Options: Yes / No
8	Have you utilized simulation training or skills lab training during this time period?	Options: Yes / No
9	Are you concerned about completing your required case numbers due to the COVID19 pandemic?	Options: Yes / No

PGY, post-graduate year. ACGME, Accreditation Council for Graduate Medical Education. COVID-19, coronavirus disease 2019. ICU, intensive care unit.

Vascular senior trainees (VST) were defined as PGY 4 to PGY 7. Vascular junior/midlevel residents (VJMR) were defined as PGY 1 to PGY 3. Analysis of the comparison of cases was performed with student t-test. Survey data was evaluated with descriptive statistics. All analysis was performed using SAS 9.4 (Cary, NC). This study was approved by the Institutional Review Board of all 3 facilities. Approval/Reference numbers are as follows: HS#: 20-00718, GCO#1: 20-1300(0001) ISMMS. Consent was not required for this study, given it was completely voluntary and there was no identifying information.

## Results

**Figure 1.**
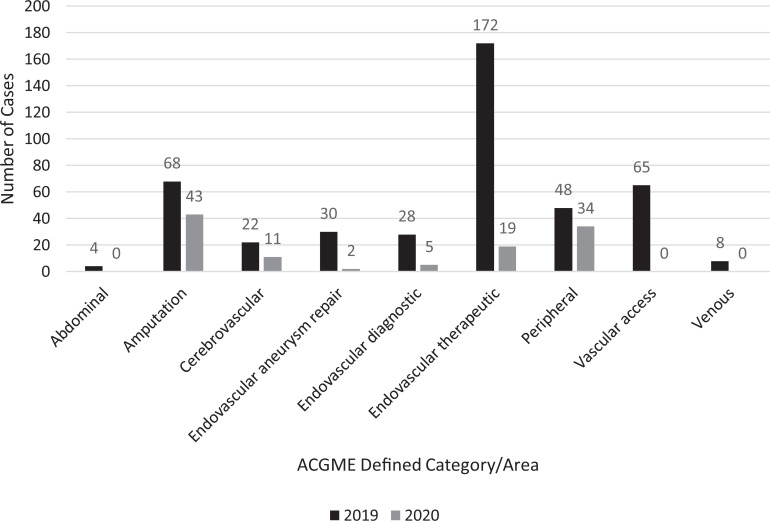
Comparison of surgical volume from March 26 to April 25 of 2019 and 2020. ACGME, Accreditation Council for Graduate Medical Education.

The total number of cases for all 3 health systems for the PPP was 114 in 2020 with 445 cases during the corresponding time period in 2019, resulting in a 74% reduction (Figure 1). For the PRE, there was a 12% decrease from 635 cases in 2019 to 560 cases in 2020. Case mix for PPP compared to the same time period in 2019 is displayed in Table 2. In 2019, the most frequent cases performed were endovascular therapeutic cases whereas in 2020, the most frequent cases performed were amputations. During PPP, venous, access, and abdominal cases all dropped down to 0. The decrease in cases was similar for both VST (77% reduction) and VJMR (75% reduction) (Table 3). There was a similar reduction in vascular surgical case volume of rotating general surgery residents (Table 3).

**Table 2.** Frequency of Cases by ACGME Category and Year for March 26 to April 25.

ACGME Category	Frequency in 2019	Frequency in 2020
Amputation	15.2%	37.7%
Cerebrovascular	4.9%	9.6%
Endovascular therapeutic	38.6%	16.6%
Endovascular diagnostic	5.1%	3.8%
Peripheral	10.7%	29.8%
Vascular access	14.6%	0%
Venous	1.8%	0%
Abdominal	0.9%	0%
Endovascular aneurysm repair	6.7%	1.7%

ACGME, Accreditation Council for Graduate Medical Education.

**Table 3.** Cases by Trainee PGY Level From March 26 to April 25 in Both 2019 and 2020.

PGY Level	Number of Cases 2020	Number of Cases 2019
VS Seniors trainees (PGY 4 to 7)	72	316
VS Midlevel and junior residents (PGY 1 to 3)	27	108
GS Senior trainees (PGY 4 and 5)	12	76
GS Midlevel and junior residents (PGY 1 to 3)	25	209

PGY, Post-Graduate Year. VS, Vascular Surgery. GS, General Surgery.

**Figure 2.**
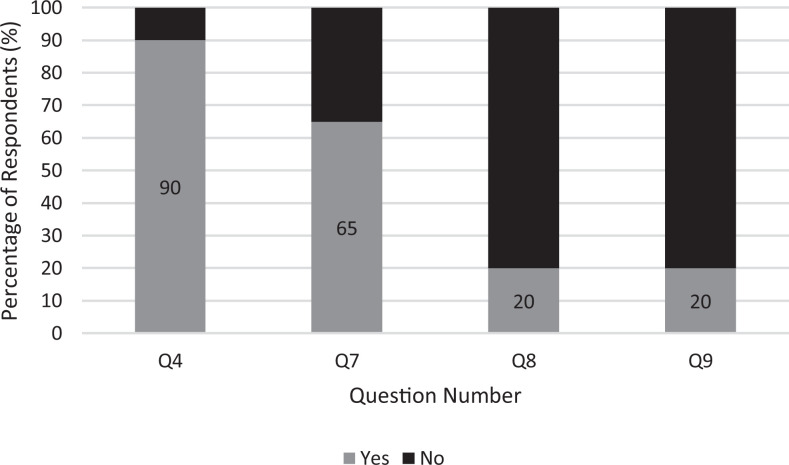
Survey responses to selected questions. Q4–question 4 (emergency surgery participation). Q7–question 7 (line placement). Q8–question 8 (simulation or skills lab use). Q9–question 9 (concern about case numbers).

Of the 26 vascular fellows and residents in the 3 programs, 20 completed the survey (77% response rate). VST constituted 50% of respondents with the remainder VJMR (Table 4). Of survey respondents, only 40% of trainees had met the minimum ACGME core case requirements for graduation and 20% expressed concern about meeting requirements. Of these concerned trainees, 3 (60%) were VJST and 2 (40%) were VST. The trainees that met requirements were VST. Of VST, 30% of the 10 respondents reported having met case requirements and 30% of the 10 were additionally worried about not meeting case number requirements. Of all the respondents, 55% had been deployed to COVID-19 units (30% intensive care units, 5% to the emergency room, and 20% were deployed to unspecified or mixed units). Of trainees that participated in the survey, 90% had performed emergency surgery, 65% were involved in central line placement and 20% participated in simulation training during the pandemic (Figure 2). Only 8 of the survey respondents indicated that they had taken medical leave due to COVID-related illness (30%), but of these 6 (75%) were away for 2 weeks or more.

**Table 4.** Post-Graduate Level and Number of Survey Participants.

PGY Level	Number (%)
1	4 (20%)
2	3 (15%)
3	3 (15%)
4	2 (10%)
5	3 (15%)
6	4 (20%)
7	1 (5%)

PGY, Post-Graduate Year.

## Discussion

There was a 74% reduction in vascular surgery case volume during the COVID-19 pandemic. The majority of survey respondents indicated involvement in traditionally non-vascular nonsurgical roles such as in central line placement of critically ill patients or in the medical management of patients in COVID units. During the study period, there were no vascular access or venous cases performed but there was an increase in the number of amputations in 2020 compared to the prior year. Despite this shift in clinical function and surgical exposure, most survey respondents did not express concern about achieving the ACGME minimum case requirements.

Didactics evolved during the pandemic. To comply with physical distancing requirements and reductions in gatherings,^12^ didactic curricula, case presentations, journal clubs, mortality and morbidity conferences and grand rounds lectures have been transitioned to remote teleconferences. Regional and national cooperatives during this time have also developed as surgical departments nationwide turn to digital technology and online platforms in order to continue educational programming for both faculty and trainees.^13,14^ The shift to a fully remote learning environment has prompted some changes in educational approach. The “flipped classroom” model is now being increasingly employed.^13^ In this approach, pre-recorded lectures are provided to trainees with the scheduled time reserved for more interactive activities and discussion. Adjunctive sessions such as simulation and surgical video review have previously been described as effective modes of supplemental training.^15^

The COVID-19 pandemic led to reduction in surgical case volumes and modified learning opportunities in order to meet governmental agency guidelines regarding elective cases and social distancing.^9^ In this study, there was a significant decrease in open and endovascular aortic surgeries and an increase in amputations. Considering the reduction in open aortic cases nationally, this is concerning as surgeon volume in open aneurysm repair is correlated with successful postoperative outcomes.^16^ In contrast to the decrease in aneurysm repair, there was an increase in amputations during the PPP. Similar to our findings, Schuivens et al. observed more amputations during the COVID-19 lockdown as patients presented with more extensive ischemic disease.^17^ Unfortunately, this has shown trainees the time-sensitive nature of limb salvage. While the reduction in skills seen with decreased operating room (OR) time can be ameliorated by increased surgical skills labs,^18^ the impact of delayed care for patients has an indelible negative consequence.

This study does have several limitations. Primarily, the New York City experience with both a significantly decreased case volume and with COVID-19 cases is particularly unique and may not be comparable to other locations thereby limiting the generalizability of our findings. The small sample size and retrospective design also limit this study. Finally, this study does not account for multiple trainees scrubbed in 1 case (“double-scrubbing”) which may affect the quality of surgical training, time available to trainees, and augmentation of surgical experiences and education. However, this study does provide an initial assessment of the impact of COVID-19 on the education of vascular surgical trainees.

## Conclusions

While elective operative volume has decreased greatly, trainees appear relatively overall confident in their ability to complete operative volume requirements despite transient reassignments in clinical responsibilities. The long term-impact of the COVID-19 pandemic and the resultant changes and limitations on medical student and graduate medical education have yet to be fully elucidated. Further efforts should therefore be directed at quantifying the impact on training. However, until further studies are performed, we support the use of training supplements such as teleconferences and simulation in pandemic conditions at institutional, regional, and national levels.
